# Decreased bone mineral density in ovariectomized mice is ameliorated after subsequent repeated intermittent administration of (*R*)‐ketamine, but not (*S*)‐ketamine

**DOI:** 10.1002/npr2.12132

**Published:** 2020-08-19

**Authors:** Yuko Fujita, Kenji Hashimoto

**Affiliations:** ^1^ Division of Clinical Neuroscience Chiba University Center for Forensic Mental Health Chiba Japan

**Keywords:** (*R*)‐ketamine, bone, osteoporosis

## Abstract

**Aim:**

Depression is a common symptom in people with osteoporosis. (*R*)‐ketamine produced greater potency and longer‐lasting antidepressant‐like actions than (*S*)‐ketamine in rodents. Here, we examined the effects of two ketamine enantiomers on the reduced bone mineral density (BMD) in the ovariectomized (OVX) mice which is an animal model of postmenopausal osteoporosis.

**Methods:**

Female ddY mice were OVX or sham‐operated. Subsequently, saline (10 mL/kg/d, twice weekly), (*R*)‐ketamine (10 mg/kg/d, twice weekly), or (*S*)‐ketamine (10 mg/kg/d, twice weekly) was administered intraperitoneally into OVX or sham mice for the 6 weeks. The femur from all mice was collected 3 days after the final injection, and BMD in the femur was measured.

**Results:**

The reduction of cortical BMD and total BMD in the OVX mice was significantly ameliorated after subsequent repeated intermittent administration of (*R*)‐ketamine, but not (*S*)‐ketamine.

**Conclusion:**

The study shows that (*R*)‐ketamine can ameliorate the reduced cortical BMD and total BMD in OVX mice. Therefore, (*R*)‐ketamine would be a novel therapeutic drug for women with osteoporosis.

## Abstract 2

(*R*)‐ketamine, but not (*S*)‐ketamine, ameliorated decreased bone mineral density in ovariectomized mice. Therefore, (*R*)‐ketamine would be a novel therapeutic drug for women with osteoporosis.

## Graphical abstract

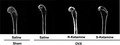

## 1 INTRODUCTION

Osteoporosis is the common chronic disease characterized by low bone mass and structural deterioration of bone tissue, leading to fragility of the bone. Depression is common in people with osteoporosis. A meta‐analysis demonstrated that bone mineral density (BMD) in patients with depression is reduced compared to subjects without depression,^1^ indicating depression as a risk factor for low BMD.^2^, ^3^, ^4^ Furthermore, the use of antidepressants is associated with osteoporotic fracture in elderly peoples.^5^, ^6^ Importantly, the use of selective serotonin reuptake inhibitors (SSRIs) is associated with decreased BMD and increased fracture risk in humans.^7^ Therefore, the development of the novel antidepressants which also have beneficial effects for osteoporosis is an unmet medical need.

(*R,S*)‐ketamine, the *N*‐methyl‐D‐aspartate receptor (NMDAR) antagonist, produces rapid‐onset and sustained antidepressant actions in treatment‐resistant patients with major depressive disorder (MDD).^8^, ^9^, ^10^, ^11^ (*R,S*)‐ketamine (Ki = 0.53 μmol/L for NMDAR) is a racemic mixture containing (*R*)‐ketamine (Ki = 1.4 μmol/L for NMDAR) and (*S*)‐ketamine (Ki = 0.30 μmol/L for NMDAR).^9^ We previously reported that (*R*)‐ketamine could produce greater potency and longer‐lasting antidepressant effects than (*S*)‐ketamine in several rodent models of depression.^12^, ^13^, ^14^, ^15^, ^16^ The detrimental side effects of (*R*)‐ketamine in rodents, monkeys, and humans were markedly lower than those of (*S*)‐ketamine.^13^, ^16^, ^17^, ^18^, ^19^ A recent open‐label study demonstrated that (*R*)‐ketamine caused rapid‐onset and sustained antidepressant effects in female treatment‐resistant patients with MDD.^19^ Collectively, (*R*)‐ketamine would be a safer antidepressant than (*R*,*S*)‐ketamine and (*S*)‐ketamine.^8^, ^9^, ^10^, ^11^, ^20^

Kadriu et al^21^ reported that infusion of (*R*,*S*)‐ketamine ameliorated abnormal levels of inflammatory bone markers in treatment‐resistant patients with MDD. Subsequently, we reported that (*R*)‐ketamine, but not (*S*)‐ketamine and (2*R*,6*R*)‐hydroxynorketamine, could ameliorate increases in inflammatory bone markers and reduced BMD in the susceptible mice after chronic social defeat stress,^22^, ^23^ indicating the beneficial effects of (*R*)‐ketamine for osteoporosis. Ovariectomized (OVX) rodents have been widely used as animal model of postmenopausal osteoporosis.^24^ However, there is no publication reporting the effects of ketamine enantiomers on the reduced BMD in OVX rodents.

In this study, we investigated the effects of (*R*)‐ketamine and (*S*)‐ketamine on the reduced BMD in OVX mice.

## 2 METHODS

### 2.1 Animals

Female adult ddY mice (n = 34, aged 12 weeks, body weight 30‐35 g) were purchased from Japan SLC, Inc (Hamamatsu, Shizuoka, Japan). Mice were housed under controlled conditions for temperature (23 ± 1°C) and humidity (55 ± 5%) with a 12‐h light–dark cycle (lights on from 7:00 to 19:00). Mice were allowed free access to food (CE‐2; CLEA Japan, Inc, Tokyo, Japan) and water. The study was approved by the Chiba University Institutional Animal Care and Use Committee (permission number: 1‐364).

### 2.2 Materials

(*R*)‐ketamine hydrochloride and (*S*)‐ketamine hydrochloride were prepared as previously reported.^12^ The dose (10 mg/kg as hydrochloride) of ketamine enantiomers was used as previously reported.^12^, ^13^, ^15^, ^22^, ^23^ Ketamine enantiomers were dissolved in saline. Other reagents were purchased commercially.

### 2.3 OVX model and repeated administration of ketamine enantiomers

**Figure 1:**
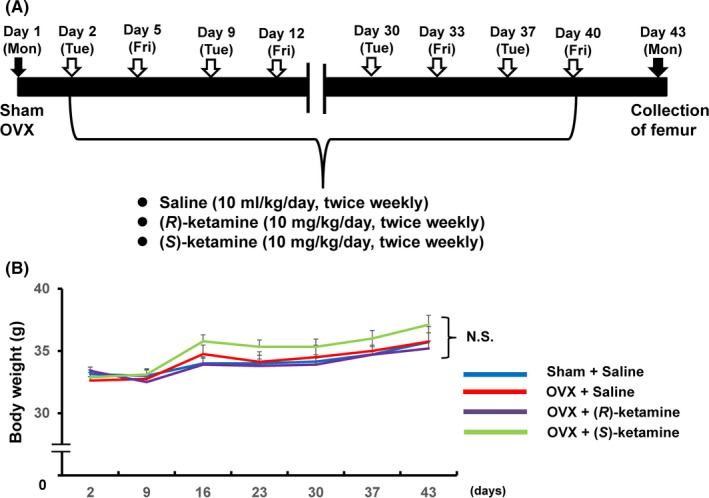
Schedule of OVX, treatment, and sample collection. A, Sham or OVX was performed on day 1 (Monday). Subsequently, saline [10 mL/kg/d, twice weekly (Tuesday and Friday)], (*R*)‐ketamine [10 mg/kg/d, twice weekly (Tuesday and Friday)], or (*S*)‐ketamine [10 mg/kg/d, twice weekly (Tuesday and Friday)] was administered i.p. to sham mice or OVX mice from day 2 to day 40. The treatment was performed for 6 wk. The femur from all mice was collected three days (day 43) after the final administration. B, Time course of body weight of mice (repeated‐measures one‐way ANOVA, *F*_6,198_ = 38.007, *P* < .001). The values represent the mean ± SEM (n = 7‐10). NS: not significance

The schedule of surgery and treatment is shown in Figure 1. Thirty‐two female mice were randomized assigned to receive OVX (n = 27) or sham surgery (n = 7) under isoflurane anesthesia. Subsequently, saline (10 mL/kg/d, twice weekly), (*R*)‐ketamine (10 mg/kg/d, twice weekly), or (*S*)‐ketamine (10 mg/kg/d, twice weekly) was administered to mice for 6 weeks. The femur from all mice was collected 3 days after the final injection (Figure 1A).

### 2.4 Determination of BMD

We quantified BMD of the femur using an experimental animal CT system (Latheta LCT‐200; Hitachi Ltd.), as reported previously.^23^, ^25^ We performed the calibration using a phantom before the measurement of the femur. Then, the femoral bones of all mice were scanned together. We determined cortical BMD, cancellous BMD, total BMD, and plane BMD using the Latheta software (version 3.40).^23^, ^25^

### 2.5 Statistical analysis

The data were shown as the mean ± standard error of the mean (SEM). The data of body weight were analyzed using the repeated‐measures one‐way analysis of variance (ANOVA). The data of BMD were analyzed using the one‐way ANOVA, followed by *post hoc* Tukey's HSD test. The *P*‐values of <.05 were considered statistically significant.

## 3 RESULTS

### 3.1 Effects of ketamine enantiomers on the reduced BMD of OVX mice

The time course of body weight was statistically significant, indicating that body weight of all groups was increased from day 2 to day 43. However, there were no changes among the four groups at each time point (Figure 1B).

**Figure 2:**
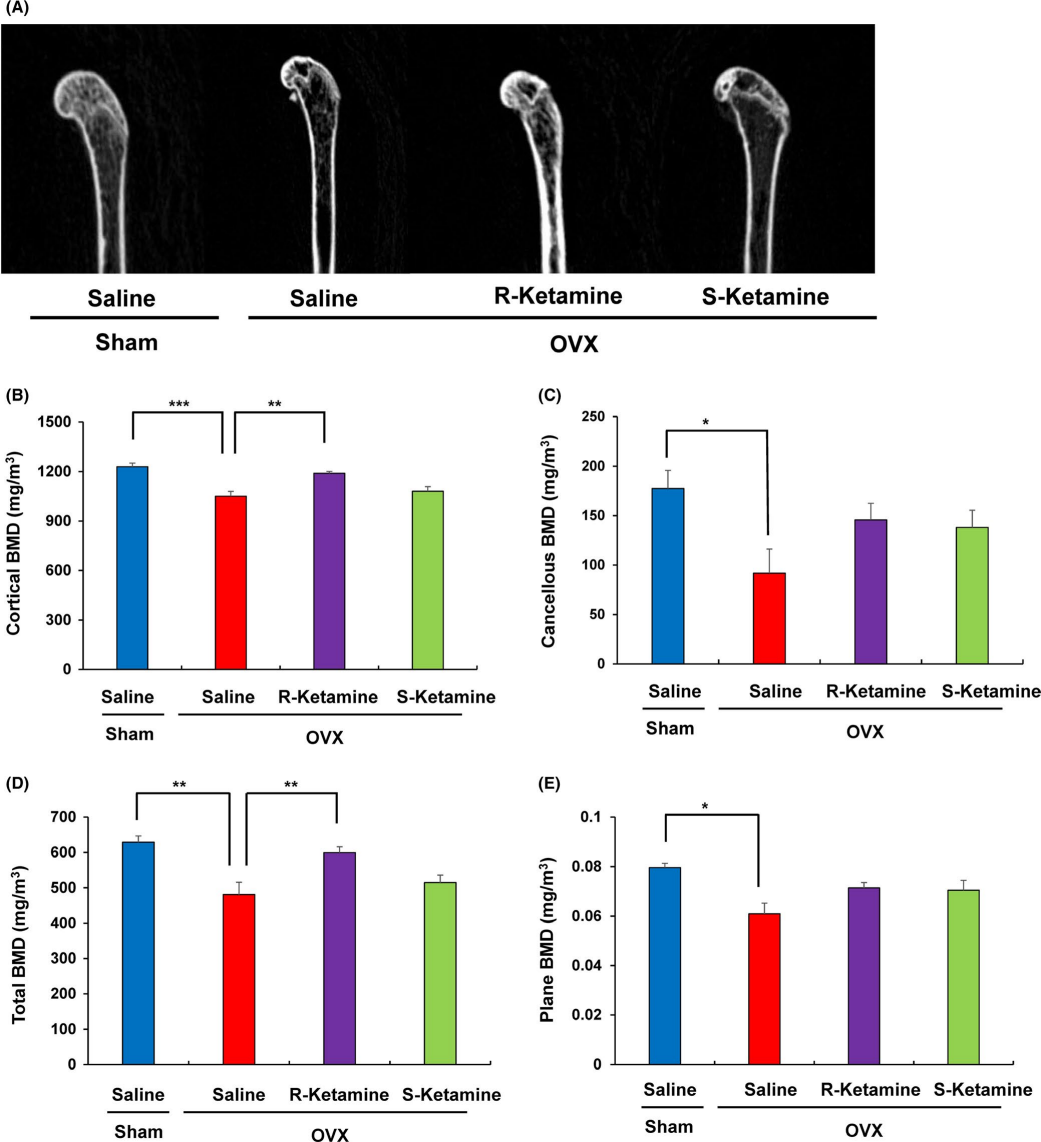
Effects of ketamine enantiomers on the reduced BMD of OVX mice. A, Representative CT of the femur from four groups. B, Cortical BMD (one‐way ANOVA: *F*_3,30_ = 13.11, *P* < .001). C, Cancellous BMD (one‐way ANOVA: *F*_3,30_ = 3.01, *P* = .046). D, Total BMD (one‐way ANOVA: *F*_3,30_ = 8.44, *P* < .001). E, Plane BMD (one‐way ANOVA: *F*_3,30_ = 4.85, *P* = .007). The values represent the mean ± SEM (n = 7‐10). **P < .01, ***P < .001 compared to saline‐treated OVX mice

Figure 2A shows the representative CT images of the femur from the four groups. One‐way ANOVA of all BMD data revealed statistical significances among the four groups. Both cortical BMD and total BMD were significantly higher in the (*R*)‐ketamine‐treated OVX group than those of saline‐treated OVX group (Figure 2B,D). In contrast, (*S*)‐ketamine did not ameliorate the reduction of cortical BMD and total BMD in the OVX mice (Figure 2B,D). Cancellous BMD and plane BMD were significantly lower in the saline‐treated OVX group than those of saline‐treated sham group. However, both ketamine enantiomers did not ameliorate the reduction of cancellous BMD and plane BMD in the OVX mice (Figure 2C,E).

## 4 DISCUSSION

In the present study, we showed that the reduction of cortical BMD and total BMD in the OVX mice was significantly ameliorated after subsequent repeated intermittent administration of (*R*)‐ketamine, but not (*S*)‐ketamine. Greater beneficial effects of (*R*)‐ketamine compared to (*S*)‐ketamine in the OVX mice were consistent with the greater potency of antidepressant‐like effects of (*R*)‐ketamine compared to (*S*)‐ketamine in rodent models of depression.^12^, ^13^, ^14^, ^15^, ^16^ Considering comorbidity of depression in patients with osteoporosis, it is possible that (*R*)‐ketamine could produce beneficial effects in female patients with osteoporosis.

Fukumoto et al^14^ reported that two ketamine enantiomers share similar pharmacokinetic profiles in rodents, indicating that the differential effects between two ketamine enantiomers for the reduced BMD in the OVX mice are not due to differences in their pharmacokinetic profiles. Therefore, it is unlikely that NMDAR might play a major role in the beneficial actions of (*R*)‐ketamine in OVX mice. Nonetheless, further studies investigating the molecular and cellular mechanisms underlying the beneficial effects of (*R*)‐ketamine in animal models of osteoporosis are needed.

As aforementioned in the introduction, the use of SSRIs is associated with hip fracture risk in the general elderly population. A retrospective cohort study of a 10‐year period showed that veterans with SSRI usage were associated with 56.7% more likely to suffer hip fracture and 34.6% more likely to develop osteoporosis.^26^ Collectively, it seems that SSRIs may not be suitable for elderly MDD patients with a risk for osteoporosis. Furthermore, the use of SSRI was associated with increased hip fracture risk in patients with hemodialysis (adjusted odd ratio = 1.25).^27^ Considering the risk of SSRI use for hip fracture, (*R*)‐ketamine could be a potential alternative drug for elderly patients with osteoporosis.

In 2019, (*S*)‐ketamine nasal spray of Johnson & Johnson was approved for treatment‐resistant patients with MDD although several concerns were addressed.^28^, ^29^ In contrast, (*R*)‐ketamine did not cause psychotomimetic and dissociative side effects in healthy control subjects, whereas the same dose of (*S*)‐ketamine produced these side effects in the healthy subjects.^18^ In addition, a pilot study showed low incidence of dissociation in treatment‐resistant MDD patients after a single administration of (*R*)‐ketamine.^19^ Thus, it is recognized that detrimental side effects (ie, psychotomimetic effects and dissociation) of (*R,S*)‐ketamine are associated with (*S*)‐ketamine.^30^ Taken together, it is likely that (*R*)‐ketamine is a potential therapeutic drug for osteoporosis.

In conclusion, this study suggests that (*R*)‐ketamine, but not (*S*)‐ketamine, significantly ameliorated the reduced BMD in OVX mice. Therefore, it is likely that (*R*)‐ketamine would be a potential therapeutic drug for patients with osteoporosis.

## CONFLICT OF INTEREST

Dr Hashimoto is the inventor of filed patent applications on “The use of *R*‐ketamine in the treatment of psychiatric diseases” and “Preventive or therapeutic agent and pharmaceutical composition for inflammatory diseases or bone diseases” by the Chiba University. Dr Hashimoto also declares that he has received research support and consultant fees from Dainippon Sumitomo, Otsuka, and Taisho. The other author declares no conflict of interest.

## AUTHOR CONTRIBUTIONS

KH is responsible for the design of the research and experiment and supervised the experimental analyses. KH wrote the paper. YF performed the experiments. YF analyzed the data. All authors read and approved this paper.

## APPROVAL OF THE RESEARCH PROTOCOL BY AN INSTITUTIONAL REVIEWER BOARD

N/A

## INFORMED CONSENT

N/A

## REGISTRY AND THE REGISTRATION NO. OF STUDY/TRIAL

N/A

## ANIMAL STUDIES

All animal experiments were approved by the Animal Care and Use Committee of Chiba University.

## Supporting information

Supplementary Material

## Data Availability

The data that support the findings of this study are available in Appendix S1 of this article.
